# Recombinant human follicle-stimulating hormone produces more oocytes with a lower total dose per cycle in assisted reproductive technologies compared with highly purified human menopausal gonadotrophin: a meta-analysis

**DOI:** 10.1186/1477-7827-8-112

**Published:** 2010-09-16

**Authors:** Philippe Lehert, Joan C Schertz, Diego Ezcurra

**Affiliations:** 1 Department of Statistics, Faculty of Economics FUCAM, Louvain Academy, 151, chaussée de Binche, B-7000 Mons, Belgium; 2 EMD Serono, Inc. (an affiliate of Merck KGaA, Darmstadt, Germany), One Technology Place, Rockland, MA 02370, USA; 3 Merck Serono S.A. - Geneva (an affiliate of Merck KGaA, Darmstadt, Germany), 9 Chemin des Mines, Geneva, CH-1202, Switzerland

## Abstract

**Background:**

Human menopausal gonadotrophins and recombinant human follicle stimulating hormone are the two main gonadotrophin products utilized for controlled ovarian stimulation in assisted reproductive technologies. In this meta-analysis, the number of oocytes was designated as the most relevant endpoint directly resulting from ovarian stimulation, and therefore where the drug effect may be estimated with the best sensitivity.

**Methods:**

All published randomized controlled trials on ovarian stimulation comparing the two gonadotrophin products were evaluated. Internal validity was determined using Chalmers' validated scale. If trials did not meet the established quality criteria, a sensitivity analysis assessed the stability of the results. The comparison of continuous variables was conducted following the weighted mean difference and the standardized mean difference (Cohen's effect size) with the random model. Given the known relationship of baseline conditions on treatment endpoints, results were adjusted for age, body mass index and type of infertility.

**Results:**

Sixteen studies involving 4040 patients were included. Treatment with human menopausal gonadotrophins resulted in fewer oocytes (-1.54; 95% CI: -2.53 to -0.56; P < 0.0001) compared to recombinant human follicle-stimulating hormone. When adjusting for baseline conditions, the mean difference estimate was -2.10 (95% CI: -2.83 to -1.36; P < 0.001). A higher total dose of human menopausal gonadotrophin was necessary (mean difference, 235.46 IU [95% CI: 16.62 to 454.30; P = 0.03]; standardized mean difference, 0.33 [95% CI: 0.08 to 0.58; P = 0.01]). The pregnancy absolute risk difference (RD [hMG-r-hFSH]) for fresh transfers was 3% (P = 0.051), and the relative risk 1.10 (P = 0.06). When adjusted for baseline conditions, the relative risk was 1.04 (P = 0.49) and absolute difference was 0.01 (P = 0.34), respectively.

**Conclusions:**

Because baseline conditions are predictive of outcome, meta-analytic results are more sensitive when these variables are considered. Using an endpoint closely associated with the stimulation period, sufficient sensitivity is achieved to compare gonadotrophin treatments. As the largest meta-analysis published to date on this subject, treatment with human menopausal gonadotrophins is characterized by fewer oocytes and a higher total dose. When considering only fresh transfers, pregnancy rates were similar.

## Background

Gonadotrophin products utilized in ovarian stimulation are derived from urinary or recombinant sources. Urinary products include human menopausal gonadotrophins (hMG, highly purified [HP-hMG]), urinary follicle stimulating hormone (u-FSH) and human chorionic gonadotrophin (hCG). The recombinant gonadotrophin products are recombinant human follicle stimulating hormone (r-hFSH), recombinant human luteinizing hormone (r-hLH) and recombinant human chorionic gonadotrophin (r-hCG).

r-hFSH and hMG are two of the gonadotrophin products primarily used for controlled ovarian stimulation (COS) in Assisted Reproduction Techniques (ART), including *in vitro *fertilization (IVF) and intracytoplasmic sperm injection (ICSI). Although both hMG and r-hFSH have been shown to be effective, a number of studies have further compared their safety and clinical effectiveness [1-6]. r-hFSH is free from urinary protein contaminants, with less immunogenic potential than the urinary-derived medication, and *a priori *may be preferable from a safety standpoint [7-9]. However, the question regarding whether r-hFSH is preferred from the clinical perspective is the topic of ongoing debate [10-12].

In terms of its primary constituents, hMG contains both FSH and LH activity (in the form of LH and hCG, which have short- and long half-lives, respectively). According to the prevailing hypotheses, the beneficial effect of exogenous LH activity in the form of hCG may result in differences in embryo quality and endometrial receptivity, providing higher live birth rates than r-hFSH in women undergoing ovarian stimulation for ART utilizing a long gonadotrophin-releasing hormone agonist (GnRH-a) protocol [1,2]. In contrast, other authors have reported better COS outcomes with r-hFSH in terms of a lower total r-hFSH dose compared with urine-derived gonadotrophins, and an increased number of follicles, oocytes, embryos and/or pregnancies [3-6].

The comparison of hMG and r-hFSH has been evaluated in various randomized controlled trials (RCTs), retrospective studies, and meta-analyses. In their meta-analysis published in 2003, Al-Inany compared r-hFSH with urine-derived FSH products (hMG, purified FSH [FSH-P] and highly purified FSH [FSH-HP]) in IVF/ICSI cycles using a long GnRH-a protocol [13]. In four of the studies identified, the comparison of hMG (n = 603 cycles) versus r-hFSH (n = 611 cycles) was limited to clinical pregnancy per started cycle, with no significant difference found between the two treatments (odds ratio [OR] 0.81; 95% confidence interval [CI]: 0.63 to 1.05; *P *= 0.11) [14-17].

Westergaard compared hMG and r-hFSH using the same studies as Al-Inany, and added four other trials [18-22]. No differences were found for ongoing pregnancy or live birth per woman (OR 1.27; 95% CI: 0.98 to 1.64), however borderline significance was observed for some secondary outcomes in favour of both hMG and r-hFSH depending on the endpoint. In 2005, Al-Inany updated their 2003 sub-group analysis of four RCTs using the long protocol, adding two new studies for a total of 686 patients treated with hMG and 678 treated with r-hFSH [23-25]. The pregnancy rate was higher with hMG (OR 1.27; 95% CI: 1.00 to 1.62).

Including only twelve trials from a selection of twenty-one potentially eligible RCTs, Al-Inany and colleagues published a third meta-analysis in 2008 [26]. To the meta-analysis published in 2005, five additional trials were added totaling 1453 hMG cycles and 1484 r-hFSH cycles; live birth rate was selected as the primary outcome [5,22,26-29]. A significantly higher live birth rate was found for hMG (OR 1.2; 95% CI: 1.01 to 1.42; *P *= 0.04) while ovarian hyperstimulation syndrome [OHSS] rates were not significantly different (OR 1.21; 95% CI: 0.78 to 1.86; *P *= 0.39).

In their 2008 publication, Coomarasamy updated prior reviews, identifying fifteen relevant RCTs using the long GnRH-a protocol, but selecting only seven as meeting the eligibility criteria for the review [5,14-17,24,25,30]. For the primary endpoint, live birth per woman randomized, a significant increase was found in favour of hMG (relative risk [RR] 1.18; 95% CI: 1.02 to 1.38; *P *= 0.03). Reconsidering their 2008 analysis, Al-Inany and colleagues re-evaluated a subset of studies in their 2009 publication, with the aim of determining if the method of fertilization might influence the outcomes of patients receiving HP-hMG or r-hFSH [31]. Based on the subset of six studies and 2371 patients, the ongoing pregnancy/live birth rate did not differ significantly but demonstrated borderline significance for improvement with HP-hMG (OR 1.19; 95% CI: 0.98 to 1.44; *P *= 0.08) [5,14,25,27,28,32]. However, in IVF cycles this difference was significantly higher in favour of HP-hMG (OR 1.31; 95% CI: 1.02 to 1.68; *P *= 0.03) although not so for ICSI cycles (OR 0.98; 95% CI: 0.70 to 1.36; *P *= 0.89).

The results from these meta-analyses present an impression of heterogeneity and point to important issues related to the most appropriate endpoint and the difficulty in aggregating studies. As to the former point, it is our position that the initial analytic approach should focus on the number of oocytes retrieved in each treatment group, because this is the primary goal and direct result of ovarian stimulation and it is an endpoint that is common to all ART studies. Although the live birth rate constitutes the ultimate clinical endpoint of ART, it is influenced by many variables in addition to ovarian stimulation. Notably, many clinical decisions have a substantial impact on outcome during the post-oocyte retrieval phase, in particular during the periods of embryo culture and development, the embryo transfer procedure and the luteal phase immediately thereafter. Notwithstanding, many authors designated ongoing pregnancy or live birth rates as the primary endpoint, assuming that post-randomization decisions were equally affecting the two treatment groups. Indeed, it is well known that oocyte and embryo quality influence the likelihood of achieving an ongoing pregnancy [33,34].

Regarding the latter point on the challenges of aggregating data from RCTs, it is critical to note that many of the trials were designed with a specific statistical plan and power, to demonstrate superiority, non-inferiority or failed to state these details, thereby potentially contributing to the aforementioned post-randomization variability. The influence of the clinician is further pronounced at each phase, since dose modifications, method of fertilization (conventional IVF or ICSI), and other clinical decisions may be dictated by baseline variables such as patient age, antral follicle count or day 3 serum FSH levels [33]. In spite of strict patient eligibility criteria, these factors may constitute a limitation for meta-analyses based on literature reports (MAL), where baseline demographics are only summarized for each treatment group. Undoubtedly, significant heterogeneity exists between studies; therefore using an assumption of "fixed treatment" appears *a priori *unrealistic. Indeed, as evident in the brief review above of prior meta-analyses published in this field, use of different selection criteria (e.g. studies that use long GnRH-a down-regulation, GnRH-a flare protocol or GnRH antagonist) yield different results even when a similar endpoint is utilized (e.g. ongoing pregnancy or live birth rate).

With all these considerations in mind, the objective of the current systematic review and meta-analysis was to update the comparison between hMG and r-hFSH, focusing on the number of oocytes retrieved and considering the potential impact of baseline variables. In particular, we reviewed previously published meta-analyses, examining the differences in selection and exclusion of studies, and the comparison of results. Additionally, we included recently published RCTs comparing hMG and r-hFSH.

## Methods

### Identification of literature

All publications related to RCTs comparing COS with hMG and r-hFSH were identified using the Cochrane Library's Cochrane Menstrual Disorders and Subfertility Review Group specialized register of controlled trials (from January 1995 to August 2009) and the Cochrane Central Register of Controlled Trials (from January 1998 to December 2009) as well as MEDLINE (from January 1966 to December 2009) and EMBASE (from January 2000 to December 2009) databases using the following key words and/or medical subject heading (MeSH) terminology: *follicle stimulating hormone, FSH, r-hFSH, hMG, recombinant human luteinizing hormone, recombinant hCG, OHSS, randomized controlled trial, controlled clinical*. Pharmaceutical manufacturers of fertility medications were contacted to identify additional unpublished and ongoing trials meeting the search criteria.

### Study selection and review methods

Prospective randomized or quasi-randomized controlled trials (assimilated to randomization but not strictly randomized, such as attributing treatment according to age) comparing hMG and r-hFSH for COS in both IVF and ICSI were included, irrespective of use of GnRH agonists or antagonists. Studies that included patients with polycystic ovarian syndrome (PCOS) were excluded from the main analysis, although they were included in the sensitivity analyses.

The selection of studies for inclusion in the review and data extraction were undertaken by two reviewers (D. Ezcurra, E. Varlan), with disagreements resolved by a third reviewer (F. Contard). Where published reports contained insufficient information, the authors were contacted for additional details which were used to make a decision about the trial's eligibility for inclusion. The methodological quality of unpublished reports and publications of trials was evaluated for both the quality of the trial and the details reported. The intrinsic quality of each study report was assessed using a validated scale that scores multiple aspects of the trial's experimental design, including sample size, randomization methods, methods to preserve blinding, selection and withdrawal criteria, outcome criteria, and statistical analysis; the scoring range was from 0 to 100 [35].

Assessment of internal validity was performed using the validated scale developed by Chalmers which judges appropriateness of randomization and double blinding and a description of dropouts and withdrawals by intervention group [35]. The full publications and the structured abstracts of the clinical trials not identified in the previous systematic reviews, masked as to authors, affiliation, sources of trial support, and journal of publication were distributed to three reviewers (D. Ezcurra, E. Varlan, F. Contard) for quality assessment. The arithmetic mean of the Chalmers Score was calculated and when values with differences greater than 30 were observed, consensus was reached by the reviewers. There were no major disagreements between the reviewers. Only data from unpublished or published reports of trials with an acceptable quality (Chalmers Score > 50) were used for the final analysis. When trials were eliminated by quality criteria, a sensitivity analysis was used to assess the stability of the results.

All the studies according to the aforementioned criteria were included, without restriction of language, published in peer-reviewed journals, or as meeting abstracts, or unpublished. We evaluated all reports with these conditions; however, our selection of reports was limited to those for which enough accuracy was provided on methodology.

## Statistical analysis

The primary objective of the current analysis was to compare gonadotrophin treatments administered during the period of ovarian stimulation. Accordingly, we selected the number of oocytes as the primary endpoint, an outcome directly following the COS period, as the most appropriate measure, since this is the point of least influence by the clinician in an RCT. Furthermore, the number of oocytes is the most common result reported in the trials. Secondary endpoints were also considered, including the total dose of gonadotrophins, clinical pregnancy rate (CPR), OHSS, and live birth rate when documented in the publication.

The number of oocytes was considered to follow a pattern of normal distribution. Differences over all the studies were pooled and weighted by the inverse of their variance (weighted mean difference method). For binary variables (OHSS or CPR), we estimated the relative risk (RR) and the absolute risk difference (RD) to estimate the number needed to treat (NNT), since these two statistics are considered more clinically intuitive than odds ratios [36]. The comparison of continuous variables (number of oocytes, gonadotrophin dosage) was conducted following both the weighted mean difference and the standardized mean difference (Cohen's effect size). As the number of trials was limited, and had unequal sample sizes, we systematically used the random effects model, which is much more adaptable as shown in Brockwell [37]. The fixed effects model was used only for sensitivity purposes.

Due to the analytic approach employed, our meta-analysis favoured the inclusion of a maximum number of studies, excluding only studies for which internal validity was considered weak or insufficiently documented. Evidence of superiority of one treatment was accepted when the results of the main analysis and the sensitivity analyses were consistent.

### Consideration of baseline factors

Consistent research has demonstrated that ART outcomes, particularly end-stage pregnancy endpoints, are strongly affected by certain baseline conditions [38-40]. Individual patient data was not available for our analysis however known factors predictive of number of oocytes and CPR were used in an attempt to adjust for these conditions. Among literature published on predictive factors, Howles and colleagues examined factors related to the number of oocytes and identified age, basal FSH, body mass index (BMI), and number of follicles as the determinant predictors from a pool of 1378 patients [38]. For pregnancy rates, Lintsen and colleagues provided evidence of the key factors of age and cause of infertility, in particular primary infertility [39]. While a majority of the trials reported non-significant differences of baseline demographics, some trials noted significant differences. The absence of a significant difference in baseline variables in a small trial does not rule out the potential impact of even a slight imbalance of these factors on treatment outcomes. Therefore an adjustment for baseline correction was performed by correcting the observed value of the tested drug by its estimated marginal value (EMV) at the baseline conditions for the control treatment.

For continuous variables, by designating *y *as the primary endpoint, *x_1_,.x_k _*as the baseline predictors, and *a_0_,a_1_,.a_k _*estimated by a general linear model, the prediction model found in the literature and the estimated marginal mean (EMM) Y_γ _of the tested drug for baseline conditions determined by the control statement γ_1_, γ_k _are:

(1) Y = a_0 _+ ∑ a_i _x_i_; and

(2) Y_γ _= a_0_+ ∑ a_i _γ_I_; thus

(3) from (1) and (2), Y_γ _= Y + ∑ a_i _(x_i _-γ_i_) [41].

For binary variables like CPR, the same calculation was based on logarithmic transformations, where the *a_i _*is the estimated log (hazard ratios):

(1) Log(Y) = a_0 _+ ∑ a_i _x_i_; and

(2) Log(Y_γ_) = a_0_+ ∑ a_i _γ_I_; thus

(3) from (1) and (2), Y_γ _= Y exp (∑ a_i _(x_i _-γ_i_)).

For the number of oocytes, age and BMI were considered as the predictive factors according to Howles [38]. However other variables such as number of follicles were not documented in the literature and therefore were not included in our analysis. For age and BMI, the coefficients a_age _= 0.21 and a_bmi _= 0.17 were used. Moreover, when age was reported as the proportion of women > 35 years, we used a_age _= 1.5 in using the difference of the proportions. When evaluating CPR, age, primary infertility and male factor infertility were used as the predictive factors according to Lintsen, et al. [39]. For age and infertility, the estimated hazard ratios (HR), HR_age _= 0.95/year and HR_inf _= 0.9 were used. Moreover, when age was reported as the proportion of women > 35 years, a_age _= 0.5 was used for the difference of the proportions. For variables not available in between-group comparison, the equality of baseline conditions was assumed; however, the study was weighted inversely proportional to the number of documented baseline conditions.

Given the expected high number of patients and to preserve balance of both types of possible statistical errors, a *P *value of < 0.01 was considered to be significant. For statistical calculations, RevMan (Release 5.0.22, Cochrane Collaboration, Oxford, UK) and SAS 9.1 (SAS Institute, Inc, Cary, NC USA) were used.

## Results

### Main study list and sensitivity analyses

From 30 publications comparing r-hFSH versus hMG in ART, seven were meta-analyses/reviews (Al-Inany [2003, 2005, 2008, 2009], Westergaard, Coomarasamy, Afnan), four were excluded as duplicate publications (Plateau [2004, 2008], Smitz, Ziebe) and three studies were excluded because of insufficient details (Elwin [unpublished data], Loutradis, Strowitski) [1,2,13,18,23,26,30-32,42-45]. The 16 remaining studies which were truly or quasi-randomized met the inclusion criteria and were found to be of acceptable internal validity [5,14-17,19-22,24,25,27-29,46,47]. Two studies were reported only via congress abstracts (Ruvolo, Serhal) while 14 studies were published as peer-reviewed papers. A total of 4040 patients were analysed in this meta-analysis, and the primary characteristics of the included studies are summarized in Table 1.

**Table 1:** Characteristics of included studies comparing hMG versus r-hFSH

First author, year, reference number	Methods	Patient population	Interventions	Chalmers Score
Duijkers 1997 [46]	RCT, allocation method not specified	Female patients with tubal pathology or unexplained infertility, ages 20 to 40 years	GnRH-a for 14 days then HMG versus r-hFSH 150 IU daily	58

Jansen *et al*. 1998 [19]	RCT, assessor-blind; allocation by number from randomization list that corresponded to medication box	Normo-ovulatory females, ages 18 to 39 years; excluded endocrine-related causes, including PCOS, and male infertility	HMG versus r-hFSH 150-225 IU daily for 4 days then adjusted	72

Kornilov *et al*. 1999 [20]	RCT, allocation by randomization method not provided	Female patients undergoing IVF	GnRH-a long protocol then hMG versus r-hFSH 150-300 IU daily for 5 days then adjusted	67

Serhal *et al*. 2000 [21]	Pseudo-randomised, open-label, single-centre study. Allocation by alternating weeks.	Couples with infertility due to tubal factor or unexplained, endometriosis and male factor infertility allowed, female age < 40 (mean 34, SD 4.4) yrs, BMI < 30	GnRH-a long protocol then hMG versus r-hFSH 150-300 IU daily for 5 days then adjusted	60

Ng *et al*. 2001 [16]	RCT, allocation by computerized randomization in sealed envelopes	Normo-ovulatory females, age < 40 years; severe male factor requiring ICSI	GnRH-a long protocol then hMG versus r-hFSH 300 IU for first 2 days, then 150 IU daily	56

Strehler *et al*. 2001 [22]	RCT, allocation by computerized randomization	Unselected female population that did not specifically exclude PCOS, age ≤ 40 years.	GnRH-a short protocol then hMG versus r-hFSH 150-450 IU daily	67

Westergaard *et al*. 2001 [17]	RCT, allocation by computerized randomization	Normo-ovulatory females, age < 40 years; excluded endocrine-related causes, including PCOS	GnRH-a long protocol then hMG versus r-hFSH 225 IU daily for 7 days then adjusted	64

Gordon *et al*. 2001 [15]	RCT, assessor-blinded; allocation by computerized randomization	Normo-ovulatory females, ages 20 to 39 years; excluded endocrine-related causes, including PCOS, and male infertility	GnRH-a long protocol then hMG versus r-hFSH 225 IU daily for 5 days then adjusted	63

European and Israeli Study Group 2002 [14]	RCT, allocation by computerized randomization list in blocks of four	Normo-ovulatory females, ages 18-38 years; excluded endocrine disorders, including PCOS	GnRH-a long protocol then hMG versus r-hFSH 225 IU daily for 5 days then adjusted	71

Kilani *et al*. 2003 [25]	RCT, allocation by randomization sequence	Normo-ovulatory females with no PCOS or endometriosis	GnRH-a long protocol then hMG versus r-hFSH 150 IU daily for 14 days then adjusted	67

Balasch *et al*. 2003 [24]	RCT, allocation by computerized randomization	Normo-ovulatory females, ages 26-37 years with no PCOS	GnRH-a long protocol then hMG versus r-hFSH 150 IU daily for 14 days then adjusted	65

Rashidi *et al*. 2005 [29]	RCT, allocation by computerized randomization	Normo-ovulatory females, ages ≤ 35 years with no PCOS or endometriosis	GnRH-a long protocol then hMG versus r-hFSH 150 IU daily then adjusted	72

Andersen *et al*. 2006 [5]	RCT, allocation by computerized randomization, stratified by patient age (< 35 years, 35-37 years)	Normo-ovulatory females, ages 21-37 years; excluded PCOS, endometriosis stage III/IV, severe male factor requiring ICSI	GnRH-a long protocol then hMG versus r-hFSH 225 IU daily for 5 days then adjusted	77

Hompes *et al*. 2008 [28]	RCT, allocation by permuted blocks of random size	Unselected female population, ages 18-39 years, excluding endocrine abnormality including PCOS	GnRH-a long protocol then hMG versus r-hFSH 150 IU daily fixed dose with adjustment permitted	76

Bosch *et al*. 2008 [27]	RCT, allocation by computerized allocation	Normo-ovulatory females, ages 18-37 years, excluding PCOS	OCP pre-treatment then hMG versus r-hFSH 225 IU daily for 2 days, then adjusted; fixed GnRH-ant protocol beginning cycle day 6	57

Ruvolo *et al*. 2009 [47]	RCT, allocation by computerized allocation	Unselected IVF female population whit FSH level of < 12 IU/mL and BMI < 28 kg/m^2^	GnRH-a long protocol then hMG versus r-hFSH 225 IU daily fixed dose with adjustment permitted	52

RCT = randomized controlled trial; hMG = human menopausal gonadotrophins; r-hFSH = recombinant human follicle-stimulating hormone; PCOS = polycystic ovarian syndrome; GnRH-a = gonadotrophin releasing hormone agonist; IVF = in vitro fertilization; ICSI = intracytoplasmic sperm injection

The main analysis was based on all 16 studies (Table 1). The sensitivity analysis was carried out on two other selections: sub-group 1 was comprised of 15 studies, excluding Strehler as the study population included patients with polycystic ovarian syndrome (PCOS); and sub-group 2 consisted of 14 studies excluding those described by abstracts only (Serhal and Ruvolo) [21,22,47]. The effect size was estimated on the main analysis, however, a significant difference in favour of one of the two treatments was considered only when all three analyses were significant at the two-sided level of significance (*P *= 0.01). Pregnancy data was included by all authors; however, not all reports distinguished between clinical PR and live birth rate. For the included studies, the reported infertility diagnoses were tubal disease, male factor infertility, endometriosis and unexplained infertility, however, Ng restricted the indication for ART to male factor infertility only in their comparison between groups; that is, no specific female diagnosis was included [16].

Women over 40 years old were specifically excluded in the 16 trials. The mean age was documented per treatment arm, and although no differences were significant, some trials (in particular smaller trials) were found to have slightly different ages. In 14 out of 16 studies, the proportion of patients with primary infertility was documented (or retrieved), ranging from 40% to 90%. The demographics of the two treatment groups matched reasonably well across the 16 studies, except for Strehler's where a significant difference in the mean number (± standard deviation [SD]) of previous cycles was observed (hMG, 0.77 [0.91] versus r-hFSH, 1.15 [0.93], *P *< 0.001) [22]. Importantly, the authors suspected that the comparison of the two treatments might be influenced by this difference.

Of the 16 included studies, 13 utilized a long GnRH agonist protocol, while three studies did not [19,22,27]. Although a significant effect may be expected due to use of a GnRH agonist short protocol, oral contraceptive pill pre-treatment and/or a GnRH antagonist, such an effect was not found, likely due to lack of statistical power (data not reported).

### Risk of bias in included studies

An attempt to quantify the risk of bias was undertaken by assigning the Chalmers score, where the individual aspects of the trials' methods were aggregated (Table 2). We found an acceptable value for all the trials (Chalmers > 50), although the trials were not of the same quality, and particular concerns were noted during the scoring process, resulting in lower final scores. Trials used randomization lists or were quasi-randomized and concealment of allocation was detailed in most of the studies. No marked differences were found in comparison of baseline demographic data, in spite of visible small differences in categories such as cause of infertility or patient age, in particular with the smaller studies. In all the studies, the selection did not exactly reflect the intent-to-treat population, in particular, not all patients recruited and entering the studies were documented and analysed. As studies differed in other medications provided during the trials, potential influence of these adjunct medications in the larger studies may have influenced the results. Only a few of the reports mentioned the statistical power being tested, but it was possible to calculate the power *a posteriori*.

**Table 2:** Current and prior meta-analyses comparing hMG versus r-hFSH

	Al-In, 2003	Al-In, 2005	Al-In, 2008	Al-In, 2009	Coom 2008	West, 2003	Sponsor	Paper	Chalmers	n_hMG_	n_r-hFSH_
Duijkers, 1997 [46]	0	0	0	0	0	0	0	1	58	7	6
Jansen *et al*. 1998 [19]	0	1	1	0	0	1	1	1	72	35	54
Kornilov *et al. *1999 [20]	0	0	0	0	0	1	0	1	67	40	28
Serhal *et al*. 2000 [21]	0	0	0	0	0	1	0	0	60	144	94
Ng *et al. *2001 [16]	1	1	1	0	1	1	0	1	56	20	20
Strehler *et al*. 2001 [22]	0	1	1	0	0	1	0	1	67	248	259
Westergaard *et al*. 2001 [17]	1	1	1	0	1	1	1	1	64	189	190
Gordon *et al*. 2001 [15]	1	1	1	0	1	1	0	1	63	29	39
European and Israeli Study Group 2002 [14]	1	1	1	1	1	1	1	1	71	357	336
Kilani *et al*. 2003 [25]	0	1	1	1	1	0	1	1	67	50	50
Balasch *et al*. 2003 [24]	0	1	1	0	1	0	1	1	65	25	25
Rashidi *et al*. 2005 [29]	0	0	1	0	0	0	0	1	72	30	30
Andersen *et al. *2006 [5]	0	0	1	1	1	0	1	1	77	363	368
Hompes *et al. *2008 [28]	0	0	1	1	0	0	1	1	76	312	317
Bosch *et al*. 2008 [27]	0	0	1	1	0	0	1	1	57	122	126
Ruvolo *et al. *2009 [47]	0	0	0	0	0	0	0	0	52	10	19

16 studies identified from the literature search, compared with previous meta-analyses. The first 6 columns compared the referenced meta-analyses using the following definitions: *sponsor*, whether or not the study was funded by a pharmaceutical company; *paper*, whether a peer-reviewed publication or abstract only was available; *Chalmers*, the Chalmers internal validity mean score [29]. The last two columns are the sample sizes for each study.

Other sources of potential bias were also noted. Westergaard's 2001 publication did not provide actual data on any baseline conditions, stating only that the treatment groups were comparable [17]. The fact that there was no significant difference between treatment arms, particularly for small (n < 100) studies likely reflects the challenges encountered with a type-II error associated with an under-powered study. Dosage was response-driven for 13 of 16 trials with Balasch, Kilani and Duijkers as the exceptions, since a fixed dose was primarily used in their studies [24,25,46]. Consequently, these trials were not included in the estimation of total gonadotrophin dosage.

### Number of retrieved oocytes

**Figure 1:**
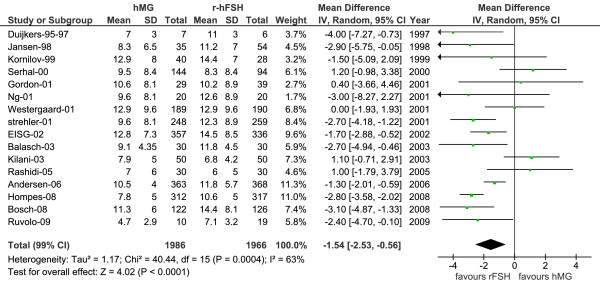
**Number of oocytes**. Number of oocytes for hMG versus r-hFSH in 16 studies (main analysis population, data reported by study authors for 3952 patients). Forest tree with mean difference using the random effects model. hMG = human menopausal gonadotrophins; r-hFSH = recombinant human follicle-stimulating hormone; EISG = The European and Israeli Study Group; SD = standard deviation; CI = confidence interval

When considering the main analysis of all 16 studies (data reported by authors for 3952 patients), significantly fewer oocytes were retrieved in the hMG treatment arm (mean 9.4 ± 6.3) compared with the r-hFSH group (mean 10.9 ± 6.6) (Figure 1 and Table 3). The mean difference was -1.54 (95% CI: -2.53 to -0.56; *P *< 0.0001) using the random model (Figure 1), and -1.74 (95% CI: -2.12 to -1.35; *P *< 0.0001) using the fixed model (Table 3, main analysis [fixed model]), with significant heterogeneity observed among the studies (I^2 ^= 63%, *P *= 0.0004). When adjusting the mean values for baseline conditions, we found a mean difference estimate of -2.10 (95% CI: -2.83 to -1.36; *P *< 0.001) (Table 3). Several secondary analyses were conducted (Table 3): a sensitivity analysis was conducted on the two aforementioned subsets, sub-group 1 and sub-group 2. Over all the alternative analyses, the mean difference varied between -1.54 and -2.10, and the difference was always highly significant (*P *< 0.001, results not shown).

**Table 3:** Number of oocytes and total gonadotrophin dose for hMG versus r-hFSH

Studied endpoint for hMG versus r-hFSH	MD	95% CI	*p *value	SDM	95% CI	*P *value
Number of oocytes						
- Main analysis	-1.54	-2.53, -0.56	< 0.0001	-0.23	-0.36, -0.10	< 0.0001
- Sub-group 1	-1.68	-2.69, -0.68	< 0.0001	-0.25	-0.38, -0.12	< 0.0001
- Sub-group 2	-1.57	-2.65, -0.49	< 0.001	-0.24	-0.38, -0.11	< 0.0001
- Main analysis (fixed model)	-1.74	-2.12, -1.35	< 0.0001	-0.26	-0.32, -0.19	< 0.0001
Number of oocytes adjusted for baseline	-2.10	-2.83, -1.36	< 0.001	-0.35	-0.47, -0.22	< 0.0001
Dosage (IU)	235.46	16.62, 454.30	0.03	0.33	0.08, 0.58	0.01

hMG = human menopausal gonadotrophins; r-hFSH = recombinant human follicle-stimulating hormone; MD = mean difference; SDM = standardized mean difference

### Total gonadotrophin dose

**Figure 2:**
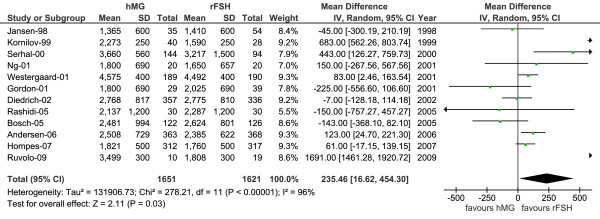
**Total gonadotrophin dose**. Total gonadotrophins dose for hMG versus r-hFSH in 14 studies (data reported by study authors for 3272 patients). Forest tree with mean difference using the random effects model. Results from Balasch, Kilani and Duijkers are not included, since a fixed dose was used in their studies [24,25,46]. hMG = human menopausal gonadotrophins; r-hFSH = recombinant human follicle-stimulating hormone; EISG = The European and Israeli Study Group; CI = confidence interval

As shown in Figure 2 and Table 3, a higher total dose of hMG was used with a MD for hMG versus r-hFSH of 235.46 IU (95% CI: 16.61 to 454.30; *P *= 0.03) and a SMD of 0.33 (95% CI: 0.08 to 0.58; *P *= 0.01]. Furthermore, we estimated the ratio of number of oocytes/1000 IU of gonadotrophin dose to be 4.39 and 5.10 for hMG and r-hFSH, respectively, with a mean difference of 0.70 oocytes/1000 IU (95% CI: 0.10 to 1.30; *P *= 0.021).

### Pregnancy rates

**Figure 3:**
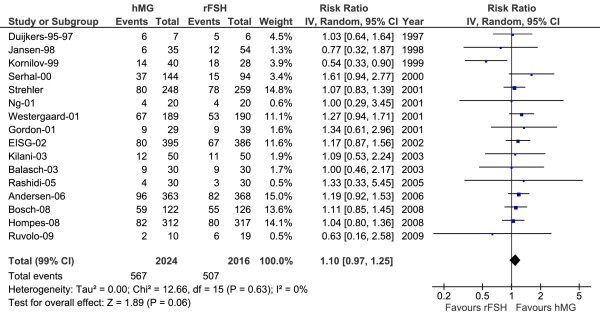
**Pregnancy rate**. Pregnancy rate for hMG versus r-hFSH in 16 studies (main analysis population, data reported by study authors for 4040 patients). Forest tree with random model and relative risk. hMG = human menopausal gonadotrophins; r-hFSH = recombinant human follicle-stimulating hormone; EISG = The European and Israeli Study Group; CI = confidence interval

Figure 3 and Table 4 present the findings of the pregnancy rates analysis for hMG versus r-hFSH. The absolute risk difference (RD) for hMG minus r-hFSH was 0.03 (95% CI: -0.01 to 0.07; *P *= 0.051) and a relative risk (RR) was found of 1.10 (95% CI: 0.97 to 1.25; *P *= 0.06). There were few indices of heterogeneity thus the effect appears homogeneous among all the studies (*P *= 0.99). When adjusting for baseline conditions, the RR for hMG versus r-hFSH was 1.04 (95% CI: 0.89 to 1.15; *P *= 0.49) and an absolute RD was 0.01 (95% CI: -0.02 to 0.04; *P *= 0.34). Pregnancy rates were also estimated and compared in the alternative selections (sub-groups 1 and 2) for sensitivity purposes by using the relative risk (RR) and the absolute risk difference (RD) (Table 4). The difference between the two treatments was not significant in any of the comparisons undertaken.

**Table 4:** Pregnancy rate and OHSS rate for hMG versus r-hFSH

Studied endpoint for hMG versus r-hFSH	RR	95% CI	*p *value	RD	95% CI	*P *value
Pregnancy rate						
- Main analysis	1.10	0.97, 1.25	0.06	0.03	-0.01, 0.07	0.051
- Sub-group 1	1.09	0.95, 1.24	0.10	0.03	-0.01, 0.07	0.08
- Sub-group 2	1.09	0.95, 1.26	0.12	0.03	-0.01, 0.07	0.08
Pregnancy rate adjusted for baseline	1.04	0.89, 1.15	0.49	0.01	-0.02, 0.04	0.34
OHSS						
- Main analysis	1.47	0.91, 2.39	0.12	0.02	-0.00, 0.04	0.72
- Sub-group 1	1.40	0.84, 2.36	0.20	0.01	-0.00, 0.03	0.65
- Sub-group 2	1.40	0.84, 2.34	0.20	0.01	-0.00, 0.03	0.62

hMG = human menopausal gonadotrophins; r-hFSH = recombinant human follicle-stimulating hormone; OHSS = ovarian hyperstimulation syndrome; RR = relative risk; RD = absolute risk difference

### OHSS rates

OHSS rates were similar between the two groups, without any significant differences in the main analysis as well as for the sub-group results (Table 4).

## Discussion

### Methodological considerations

Combined with sensitivity analyses, our method of trial selection provided a robust number of studies (n = 16) compared to prior meta-analyses which included between 4 and 12 studies comparing hMG and r-hFSH [13,18,23,26,30,31]. As a result of the large sample size of this meta-analysis, and the fact that several endpoints were considered, the level of significance was set at *P *< 0.01. Although the random model of meta-analysis is considered more conservative it is also much more realistic. It has been shown by Brockwell that for studies of unequal sample sizes or for a small number of studies, the random model is the preferred analytic method [37].

For both pregnancy rate and number of oocytes, the prediction models of Howles and Lintsen found the coefficient of determination larger than R^2 ^= 0.10, when considering baseline variables such as age, reasons for infertility, and BMI [38,39]. Compared with these determinations, the effect size observed between the two drugs is negligible (< 0.01). It follows that in an RCT comparing the two drugs, baseline conditions may have a higher effect than treatment itself. A direct consequence is that comparison between these drugs without adjustment for baseline variables can be noticeably inaccurate and biased, depending on inevitable differences at baseline. Although a baseline comparison fails to show statistically significant differences, a discrepancy may exist which may affect the treatment outcome; this is particularly true for small trials. As a result of adjusting for baseline variables when comparing the two treatments in our analysis, the impact of these variables was reduced when considering the oocyte and pregnancy rate endpoints. Age, BMI and infertility rates were used in this study, as these were the only variables consistently reported across the majority of studies. Our attempt to adjust for baseline parameters has evident limitations, but provides a very important correction in the context of meta-analyses conducted from literature research (MAL). Obviously differences in baseline conditions may be more evident in smaller studies, but the weight of a small study may remain important and bias the results. From this perspective, we believe that (1) the simple assertion that baseline characteristics are similar between treatment groups is not sufficient evidence to conclude that the treatment groups are truly matched; (2) that more baseline conditions should be routinely reported and systematically analysed; and (3) that treatment comparisons should be routinely and systematically adjusted for key baseline parameters.

### The effect of post-randomization clinical actions

In the majority of ART studies considered in this meta-analysis, only gonadotrophin treatment during the COS period was randomized. All other decisions made by the physician or embryologist were non-randomized and were center-specific and/or patient-specific. As with baseline conditions, these post-randomization actions are expected to have a key impact on ART endpoints otherwise a patient-specific approach to treatment would not be undertaken. In this context, a "distant" endpoint such as pregnancy is most likely influenced by these actions. The number of retrieved oocytes represents the first evaluable endpoint immediately following the COS timeframe, and thus the impact of post-randomization interventions is minimal, compared with more "distant" endpoints. Although not conclusive evidence of post-randomization effects, the highly significant difference (*P *< 0.001 for both baseline adjusted and unadjusted estimates) found for the number of oocytes compared with the lack of significance (*P *= 0.06 and *P *= 0.49 for unadjusted and adjusted baseline estimates, respectively) found for the pregnancy rate suggests that the number of oocytes is less influenced by post-randomization factors thus representing smaller uncontrolled variability and a more sensitive estimate of the difference between the two treatments.

### Comparison with earlier findings

#### Number of oocytes and total gonadotrophin dose

The findings of the present meta-analysis are in agreement with previous meta-analyses regarding the number of oocytes, consistently found to be higher for r-hFSH in almost all the studies and all the meta-analyses. The estimate of effect size (-0.35, 95% CI: -0.47 to -0.22) is smaller compared with Al-Inany's findings (0.80, 95% CI: 0.56 to 1.05) [26]. We also agree with earlier reports on the total doses (IU) administered. Al-Inany estimated a lower total dose administered of -282.5 IU (95% CI: -311 to -254) for r-hFSH compared with hMG; we found a very similar value of 235.46 IU (95% CI: 16.62 to 454.30), equivalent to slightly more than three 75 IU vial equivalents [26]. Thus, our estimates confirm the higher dose administered in the hMG group, although the difference was not statistically significant. Further supporting the overall finding, the number of oocytes/1000 IU total gonadotrophin dose was 4.39 and 5.10 for hMG and r-hFSH, respectively.

#### Pregnancy rate

On the basis of the 16 studies, no significant difference for pregnancy rate was found between the two treatments (*P *= 0.06 and *P *= 0.49 for baseline unadjusted and adjusted estimates, respectively). Even following adjustment for baseline conditions, the final estimate of risk difference was 0.01 (*P *= 0.49) for hMG versus r-hFSH. Based on this finding, it seems that providing estimates in terms of odds ratios may be confusing for clinicians. In particular when the studied proportion is much higher than zero, the odds ratio will always be much higher than the relative risk. Accordingly, an odds ratio of 1.20 in fact corresponds to a relative risk of 1.09 or an absolute risk difference of 0.03. Moreover, our estimated risk difference of 0.01 found by baseline adjustment is equivalent to a number needed to treat (which assesses treatment benefit) approximately equal to 100 which may be considered as a negligible value with which to reach a conclusive decision regarding the two treatments. Given the difficulty to reach a definitive conclusion on pregnancy rate when only fresh transfers are included, and considering the significant difference found for the number of oocytes retrieved, the most relevant pregnancy endpoint may be the cumulative pregnancy rate, which combines the outcomes of embryos generated from the same COS cycle, with fresh and frozen thawed transfers.

In terms of number of oocytes retrieved and total gonadotrophin dose, the differences between the two treatments is significant, whereas this is not the case for the pregnancy rate. These findings may be attributed to the difference between the two treatments (r-hFSH and hMG) as well as heterogeneity between the studies themselves. Thus, for the number of oocytes and total gonadotrophin dose, the difference between the two treatments is favourable for r-hFSH, however, a significant variance was found in the results between the individual studies. In contrast, the pregnancy rate is almost the same among treatment groups and the between-study variation for this difference is minimal. One reason for this difference is purely statistical, in which the number of oocytes and total gonadotrophin dose are counts for which the standard deviations are often proportional to the mean and are much greater than in the case of the pregnancy rate where the proportions are close to one. Additionally, the number of transferred embryos was similar between studies.

Further, the apparent absence of heterogeneity of efficacy for the two treatments may be attributed to the much smaller contribution of r-hFSH versus hMG in terms of pregnancy rate, where post-randomization variables have a greater impact on treatment outcome. Indeed, the homogeneity of the difference between r-hFSH and hMG may be regarded as additional evidence of the difficulty in observing such an effect since the drug administered is one of many interventions that ultimately impact the treatment outcome. In contrast, the number of oocytes and total gonadotrophin dose are variables with a more causal effect with the studied treatments as their impact is assessed directly during or immediately following the stimulation period. In this regard, the effect of the gonadotrophin is important, thus significant differences may be expected.

## Conclusion

As the largest meta-analysis published on the comparison of hMG and r-hFSH for COS, based on 4040 patients in 16 RCTs, hMG produced fewer oocytes and required a higher total gonadotrophin dose compared to r-hFSH. Pregnancy rates were found to be similar, in contrast to the conclusion of several prior meta-analyses [26,30].

Baseline conditions are known to determine treatment outcome, perhaps to the same extent as treatment interventions. Accordingly, a limitation of the present meta-analysis which is inherent to all MAL meta-analyses in which individual patient data is not available, is the approximation of the influence of baseline conditions and post-randomization procedures. Consequently, due to the limitations of MAL meta-analyses, sensitivity is improved after adjustment for baseline variables, and a comparison based on "distant" endpoints such as the pregnancy rate is of limited value due to the substantial influence of post-randomization procedures. In this regard, only endpoints as close as possible of the end of the COS period appear to have sufficient sensitivity to compare stimulation treatments. A meta-analysis based on individual patient data would enhance the sensitivity of these results.

Finally, due to the small variance observed with the two treatments during COS compared with the influential effect of baseline conditions and post-randomization procedures, it remains to be clarified whether evidence collected from meta-analyses confined to RCTs may be complimented by retrospective analyses representing a more comprehensive level of treatment experience. In these conditions, it is probably better to increase the sample size by using large databases since the randomization is limited to the COS drugs, which represent a small portion of the entire treatment intervention and therefore dilute the findings of "gold standard evidence" obtained from RCTs. As this was not the aim of the current analysis, further examination of this perspective is warranted.

## Competing interests

The meta-analysis was supported by Merck Serono, S.A., Geneva. DE and JCS are employees of Merck Serono S.A. - Geneva or EMD Serono, Inc. PL is a paid consultant with Merck Serono S.A. - Geneva.

## Authors' contributions

PL, DE, and JCS contributed to the writing and editing of the manuscript. All authors reviewed the manuscript during its development and approved the final version for submission.
